# A methodology for the generation and non-destructive characterisation of transverse fractures in long bones

**DOI:** 10.1016/j.bonr.2018.04.007

**Published:** 2018-04-25

**Authors:** Fernando Y. Zapata-Cornelio, Zhongmin Jin, David C. Barton, Alison C. Jones, Ruth K. Wilcox

**Affiliations:** a Institute of Medical and Biological Engineering, School of Mechanical Engineering, University of Leeds, United Kingdom; b School of Mechanical Engineering, Southwest Jiaotong University, China

**Keywords:** Roughness, Fracture, Micro computed tomography, Femur, Computer modeling, Surface characterisation

## Abstract

Long bone fractures are common and although treatments are highly effective in most cases, it is challenging to achieve successful repair for groups such as open and periprosthetic fractures. Previous biomechanical studies of fracture repair, including computer and experimental models, have simplified the fracture with a flat geometry or a gap, and there is a need for a more accurate fracture representation to mimic the situation *in-vivo*. The aims of this study were to develop a methodology for generating repeatable transverse fractures in long bones *in-vitro* and to characterise the fracture surface using non-invasive computer tomography (CT) methods. Ten porcine femora were fractured in a custom-built rig under high-rate loading conditions to generate consistent transverse fractures (angle to femoral axis < 30 degrees). The bones were imaged using high resolution peripheral quantitative CT (HR-pQCT). A method was developed to extract the roughness and form profiles of the fracture surface from the image data using custom code and Guassian filters. The method was tested and validated using artificially generated waveforms. The results revealed that the smoothing algorithm used in the script was robust but the optimum kernel size has to be considered.

## Highlights

• A new method to generate and quantify long bone fracture surface geometry is proposed.
• Reliable transverse fractures were generated and imaged using microCT.
• A non-destructive method to characterise the surface from the images was developed
• Validation of the method using benchmark waveforms showed the method to be robust
• By tuning the filtering conditions, specific characteristics of the surface can be extracted.

## 1 Introduction

Long bone fractures are common, with incident rates of 400 per 100,000 population (Meling et al., 2009) reported for traumatic fractures requiring hospital management, and represent a considerable healthcare burden (Bonafede et al., 2013). Whilst treatment generally has high levels of success, some groups such as periprosthetic and open fractures remain challenging to treat and non-union or failure of the fracture fixation device can occur (Springer et al., 2003; Tsiridis et al., 2003; Tsiridis et al., 2005; Tzioupis and Giannoudis, 2007). The reported failure of these devices has triggered an increasing number of biomechanical studies to investigate the mechanics of fracture fixation either by mechanical testing or computational modeling (Dennis et al., 2000; Hoffmann et al., 2014; Lenz et al., 2014; Moazen et al., 2014; Sariyilmaz et al., 2014; Lenz et al., 2016). These studies tend to neglect the effect of the geometry and surface properties of the fracture by representing the fracture as a gap or a perfectly flat surface; however there is some evidence that the variation of these features could have a major effect on interfragmentary movement (IFM) (Mak, 2013; Leonidou et al., 2015). It is widely accepted that IFM influences the healing process (Wu et al., 1984; Goodship and Kenwright, 1985; Claes et al., 1995; Claes et al., 1997) and, to some extent, a certain degree of IFM can encourage callus formation (Bottlang and Feist, 2011). Additionally, the direction of IFM plays an important role in healing, but the significance of torsional shear on IFM is not fully clear (Yamagishi and Yoshimura, 1955; Bishop et al., 2006). Despite this, the effects of representative fracture pattern, surface properties and IFM are all omitted in studies with simplified fracture representation, and therefore there is a need for more realistic representation of fractures in both experimental and computational studies.

There have been attempts to characterise surface properties of fractured bone in the past, for example by defining a bone-to-bone coefficient of friction (CoF) (Shockey et al., 1985; Von Fraunhofer et al., 1985). However results have shown that the measurements of CoF depend on the type of bone used in the experiment and the methodology used to artificially generate the fracture; this highlights the importance of measuring the fracture surface and topographical properties on specimens that closely represent realistic fractures.

The highly irregular shape and fluid content on the fractured surface of long bones make it unsuitable for characterisation methods such as surface or optical profilometry, and whilst there have been attempts to measure the roughness using SEM, this requires coating of the surfaces which prevents subsequent mechanical characterisation (Wynnyckyj et al., 2011). High resolution peripheral quantitative computed tomography (HR-pQCT) has the potential to capture the fracture surface morphology at a relatively high resolution on whole bone surfaces non-destructively, allowing the use of the specimens for further mechanical testing in the laboratory. However, as yet, no methodology appears to have been developed for quantifying the surface topographical properties using this technique.

The generation of reproducible fractures in long bones has been the subject of several studies in the literature, using either experimental, computational or combined methods (Kraft et al., 2012; Untaroiu et al., 2013). The techniques to create such fractures have varied, and include the use of a guillotine (Bonnarens and Einhorn, 1984; An et al., 1994; Marturano et al., 2008), four-point bending (Martens et al., 1986; Bramer et al., 1998) and osteotomy (Akeson et al., 1975; Wu et al., 1984). Three-point bending experiments have been shown to produce fractures with more control over the crack location (Ekeland et al., 1981; Molster et al., 1982; Macdonald et al., 1988). Limited information has been published on devices capable of generating fractures other than in small rodents (Ekeland et al., 1981; Bonnarens and Einhorn, 1984; An et al., 1994; Marturano et al., 2008; De Giacomo et al., 2014), or in sections taken from larger mammal bones (Nalla et al., 2003), and a device that could accommodate full size larger mammal bones would be beneficial.

Hence, the aim of this study was to develop a methodology for the generation of repeatable transverse fractures in long bones *in-vitro* under high-rate loading conditions and subsequently characterise the fracture surface using non-invasive CT methods. Artificially generated fracture waveforms were also used to evaluate the appropriateness of different filters in defining the fracture roughness.

## 2 Materials and methods

### 2.1 Theoretical considerations

Based on fundamentals of fracture mechanics and bone mechanics, three parameters of interest were considered in the design of the fracture rig: impact velocity (*v*), impact kinetic energy (*E*) and “span” i.e. distance between the supports of the rig. The faster the loading regime applied to the bone, the greater the energy absorption experienced by the bone before failure (Mcelhaney, 1966; Crowninshield and Pope, 1974; Currey, 1975; Wright and Hayes, 1976; Carter and Hayes, 1977). Depending on the speed of the loading regime applied to the bone, the produced fracture can be linear (slow loading) or multifragmentary (fast loading) with segments of bone expelled from the fracture site as a result of the high strain rates (McGee et al., 2004). The kinetic energy ($E=\frac{1}{2}mv^{2}$) of the drop weight is absorbed by the bone at the fracture site and, for a given impact velocity, this energy depends on the drop mass (*m*). The rig span also plays an important role in determining the kinetic energy necessary for full fracture of the femur. By increasing the span, the energy required to generate full fracture is reduced because the bending moment at the centre of the span for a given impact load is increased.

### 2.2 Specimen preparation and fracture generation

Ten porcine legs (aged 24–26 weeks) were harvested within 24 h of slaughter and all tissue surrounding the femoral bone was removed. Each specimen was placed in a plastic sealable bag and refrigerated at 5 °C prior to testing.

**Fig. 1:**
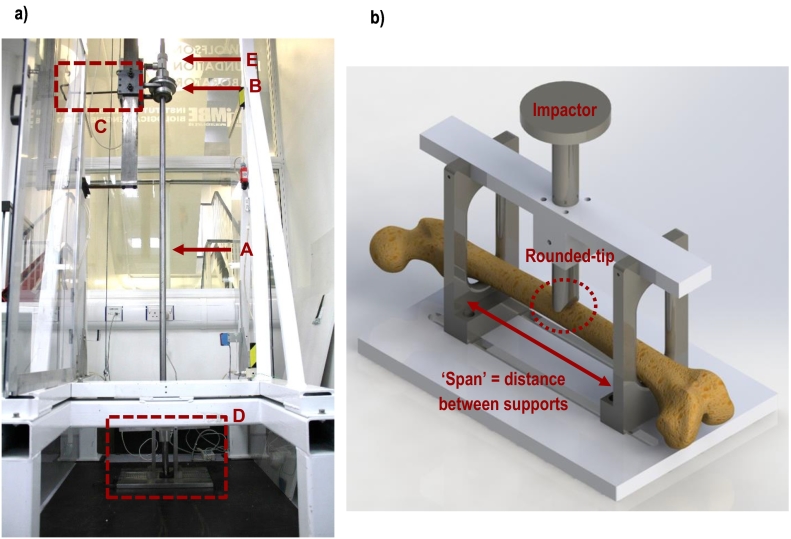
a: Drop test rig and fracture rig set-up showing A: the drop shaft, B: the drop weight, C the release mechanism, D: the mounting rig, E: the height adjustment. 1b: Close-up diagram showing the main components of the fracture rig and positioning of the specimen before fracture.

In order to generate repeatable impact fractures *in-vitro*, a specially designed fracture rig was manufactured in-house and used together with a drop test rig (Wilcox, 2002) that comprised a vertical shaft down which a mass could be released from an adjustable height (1.1 to 2.0 m). The device was manufactured using stainless steel, due to its corrosion resistance and mechanical properties. The fracture rig was designed to load the specimen under three-point bending (Fig. 1). It comprised two rounded supports on which the specimen rested which could be adjusted so that they touched the pectineal line of the lesser trochanter at the proximal end and the adductor tubercle at the distal end of the specimen, maximising span and therefore reducing the amount of energy required to produce the fracture. The impact was applied at a point midway between the supports through a round-tipped impactor of the same radius as the supports. The impactor was attached through a shaft to a plate onto which the mass fell. This produced the greatest bending moment at the mid-span position, resulting in the generation of the fracture at approximately the centre of the bone.

Following a pilot study in which the impact mass and drop height were varied, a mass of 2.5 kg and a drop height of 1.9 m (ie impact energy of 47 J) were found to most reliably generate transverse fractures and these settings were subsequently used to generate fractures in all the specimens in this study.

Following the fracture, the distal section of each specimen was imaged (peak voltage 60 kVp, current 900 μA, integration time 300 ms, voxel size 82 μm) using a HR-pQCT scanner (XtremeCT, Scanco Medical AG, Brüttisellen, Switzerland) to capture the full fracture surface and 10 to 15 mm of the bone shaft.

### 2.3 Post-processing of HR-pQCT images

The HR-pQCT images were imported into a commercial image processing software (Scan-IP, version 5.1 – Build 1012, Simpleware, Exeter, UK) and the bone was segmented using a combination of floodfill and paint-with-threshold algorithms. The segmented images were exported as TIFF files and a skeletonise function was applied (ImageJ, v 1.51a, Maryland, USA) to an image slice through the bone, slightly outside the fracture site.

Any branching lines were removed (Matlab, MathWorks, vR2012b – 8.0.0.873, Cambridge, UK) to leave an image with a single pixel-wide mid circumference of the cortex. A bespoke script in Matlab was used to project the medial circumference line onto the fracture surface as follows: each point in the skeletonised circumference was projected sequentially through the stack of binarised images until the fracture surface was reached (*i.e.* when the point lay in the segmented bone region), and the height of the projection determined from the slice number of the image reached.

**Fig. 2:**
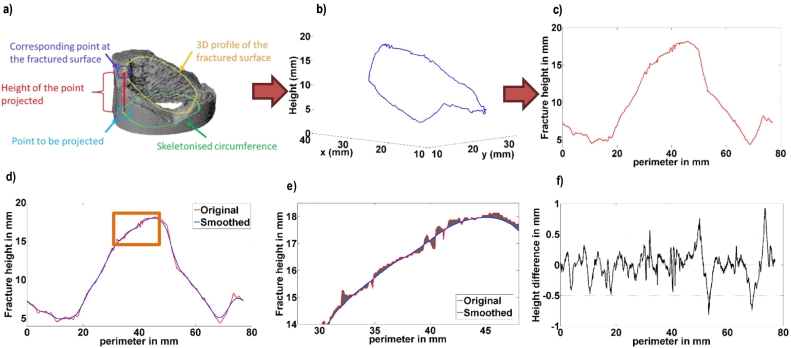
a) The projection method used to capture the fractured surface of each specimen. b) The resulting 3D profile. c) The 2D fracture profile plotted against its perimeter. d) The original 2D profile (red) and smoothed profile, defined as the “form” (blue), created using a kernel size 6.7 mm. e) A close up of the region in the orange box with the area shaded in grey representing the difference between the profiles (*i.e.* the roughness profile). f) Roughness profile: difference in height between original and smoothed profiles. (For interpretation of the references to colour in this figure legend, the reader is referred to the web version of this article.)

This process was repeated for each point in the skeletonised circumference, generating as a result a 3D profile of the fractured surface. Finally, the fracture height was plotted against distance around the circumference as a 2D ‘fracture profile’. Irregularities in the profile caused by holes or longitudinal splits in the cortex were smoothed using an automated code. The process is summarised in Fig. 2a,c,d.

### 2.4 Quantification of surface topography

A number of terms are standardly used in metrology to define the surface finish of an object, including surface roughness, waviness and form. In this manuscript, the overall (macroscale) fracture shape is referred to as the “form” and the term “roughness” is used to describe the smallest detectable surface waveforms, in the order of 100 μm, although it should be noted that their wavelengths are much larger than those usually defined as roughness when measuring surface finish in metals. Gaussian filters have historically been used in surface analysis to separate the roughness, waviness and form (Krystek, 1996; Yuan et al., 2000; Raja et al., 2002). In this study, a normalised Gaussian window function was applied to the 2D fracture profile with the aim of evaluating the smoothing process (ie. window or kernel size) and the resulting form profile.

The “roughness profile” was defined as the difference between the original fracture profile and the form profile, Fig. 2d–f, and the roughness was quantified applying Eq. 1 to the roughness profile,

(1) $$R_{a}=\frac{1}{L}\int_{0}^{L}\left|z\left(x\right)\right|\mathrm{dx}$$

where R_a_ is the roughness, L is the total distance (perimeter) of the roughness profile, and z(x) is the function that describes the roughness profile.

### 2.5 Validation

**Fig. 3:**
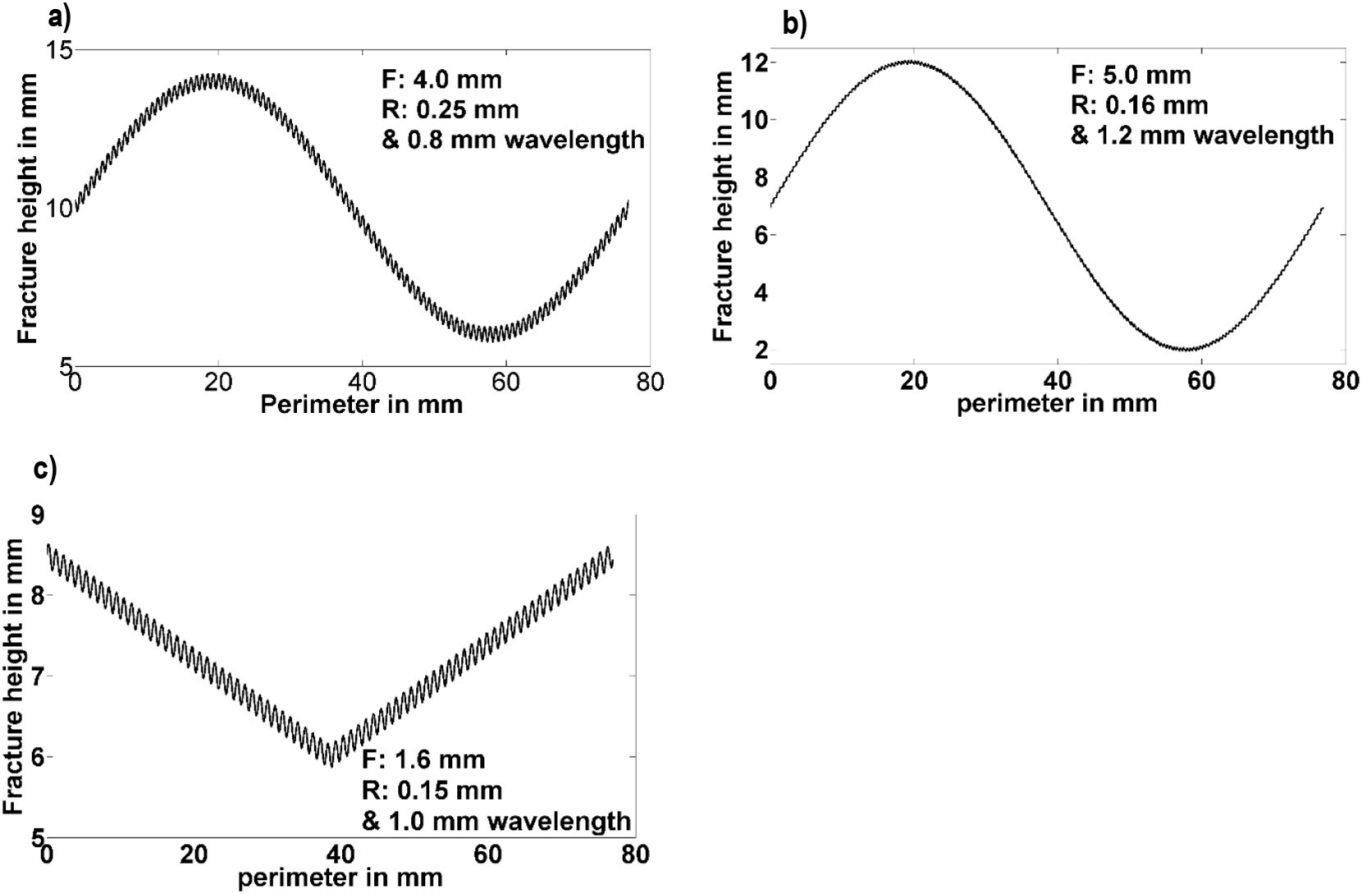
Example of fracture profiles generated to validate the smoothing process, a) F: Sine & R: Sine. b) F: Sine & R: Sawtooth. c) F: Triangular & R: Sine. F = waveform for the form, R = waveform for the roughness. Values displayed in the figures refers to amplitude, unless stated otherwise.

To validate the smoothing method and gain further insight into the effect of the kernel size in the resulting form profile, a series of three different fracture profiles were generated directly by representing the form and roughness as two components in an equation with differing shaped waveforms (1: a sinewave for both form and roughness; 2: a sinewave form and sawtooth roughness, 3: a triangular form and sinewave roughness, as shown in Fig. 3). The number of wavelengths of the form component around the circumference was varied between one and three to represent the form profiles seen experimentally.

**Fig. 4:**
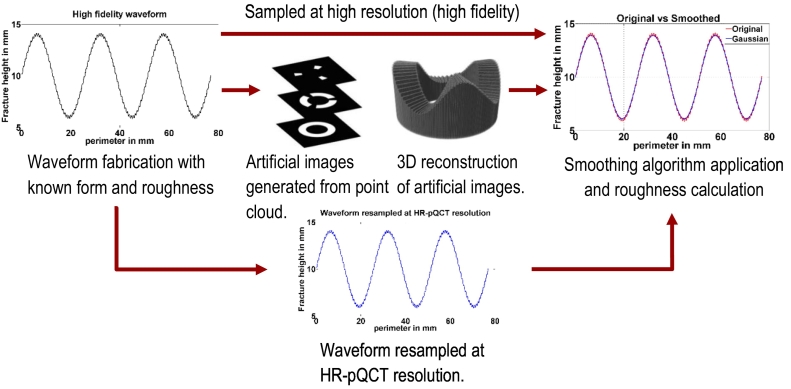
Methodology for the construction of a validation set of images.

Initially the functions were generated at a high fidelity, and then resampled to represent the resolution in the CT images, and finally images were artificially generated from the waveforms. In each step in the process the roughness was calculated using the smoothing method and different kernel sizes, and the deviation from the mathematically derived roughness was determined (Fig. 4).

## 3 Results

The data associated with this paper (images, and all processed outputs) are openly available from the University of Leeds Data Repository (https://doi.org/10.5518/344).

### 3.1 Experimental outcomes

**Fig. 5:**
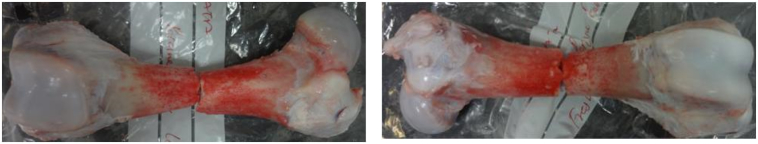
Two examples of fractures generated on femoral porcine bone.

The fracture procedure consistently created clear transverse fractures, separating the proximal and distal end of the bone without additional fragments. The AO Müller classification of fractures in long bones (Müller, 1990) defines simple transverse fractures of the femoral shaft as having a fracture line angle under 30 degrees. Using the smoothed profile, the fracture height of each specimen was calculated as the difference between the maximum and minimum points in the profile. By idealising the bone shaft as a cylinder based averaged diameter measurements, the fracture angle was then determined and found to range from 10 to 29.9 degrees (mean = 20, s.d. = 5.8 degrees). Two typical fractured specimens are shown in Fig. 5.

### 3.2 Validation outcomes

**Fig. 6:**
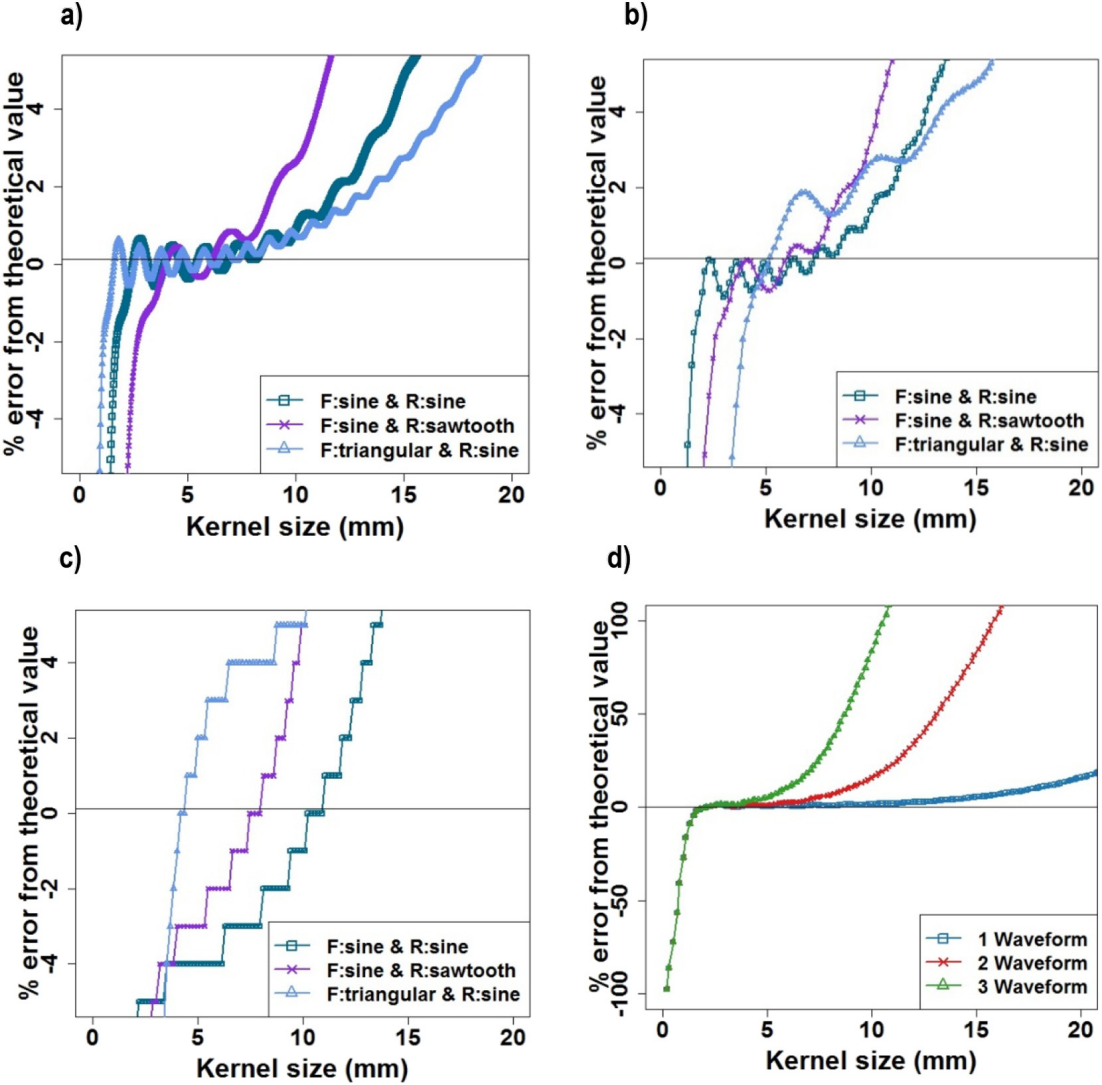
Errors from theoretical values for a) “high fidelity” waveforms with a wavelength equal to 1 circumference (i.e ~70 mm); b) waveforms with resolution equal to HR-pQCT resolution; c) waveforms captured from CT-images - conversion at 82 μm; d) example of waveforms (F:sine & R:sine) with resolution of 82 μm with one to three waveforms around the circumference. F = waveform for the form (sinewave or triangular); R = waveform for the roughness (sinewave or sawtooth).

Using the artificially generated waveforms, it was found that the roughness determined using the algorithm deviated from the mathematically generated roughness by <5% when the kernel size ranged from 4 to 8 mm for all the waveforms. This was true for the high fidelity waveforms (Fig. 6a), those resampled at the equivalent of the HR-pQCT resolution (Fig. 6b) and those derived from artificially generated images (Fig. 6c). As the number of waveforms in the fracture form profile increased, the error in calculated roughness occurred at smaller kernel sizes, with a 5% error occurring when the kernel size reached approximately ¼ of the wavelength (Fig. 6d).

### 3.3 Quantification of surface roughness

**Fig. 7:**
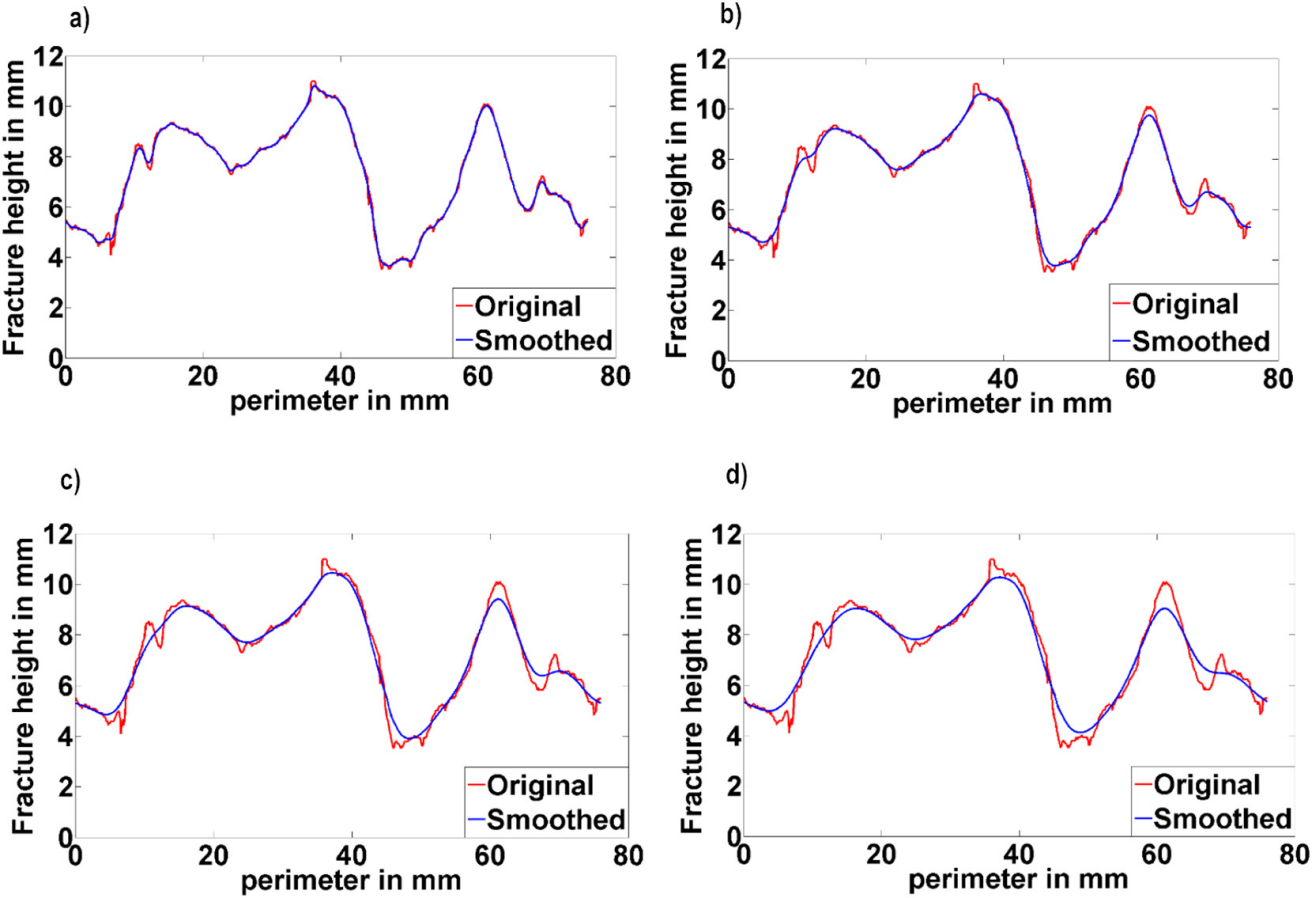
Example of a captured geometry (in red), using several kernel sizes ranging from a) 1.8 mm, b) 4.3 mm, c) 6.7 mm and d) 9.2 mm. The line in blue represents the smoothed profiles. (For interpretation of the references to colour in this figure legend, the reader is referred to the web version of this article.)

**Fig. 8:**
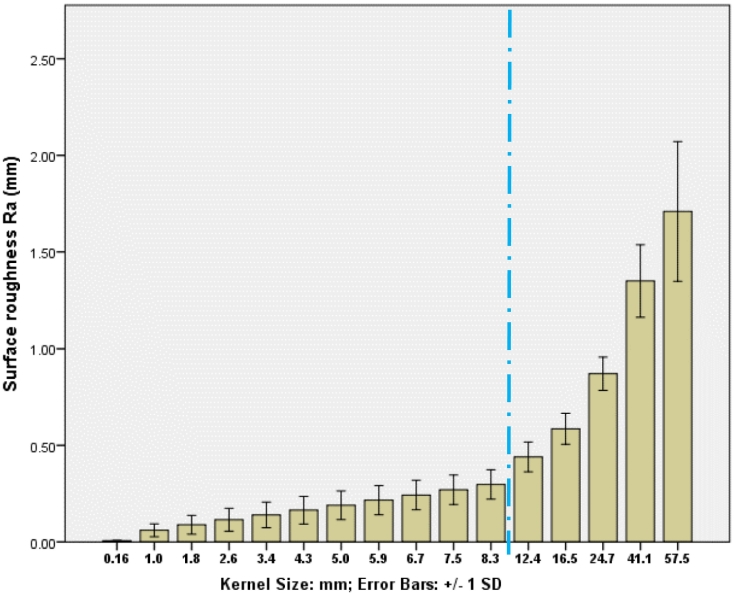
Calculated mean surface roughness using different kernel sizes across specimens (*n* = 10). The error bars indicate ±1 SD. Note that the increment in kernel size is constant below the dashed line and thereafter increases in order to show the full range of kernel sizes.

Examples of the fracture profile for one specimen are shown in Fig. 7 along with the smoothed profile generated using different kernel sizes in the Gaussian filter. The roughness values for all specimens calculated with different kernel sizes are shown in Fig. 8. It was found that the calculated roughness increased relatively slowly between kernel sizes of about 1.8 to 8.3 mm, and in this range the standard deviation was reasonably constant. At larger kernel sizes, the standard deviation increased more rapidly. These results were consistent with the results obtained in the validation section 3.1, giving confidence in the filtering method and the correct range of kernel size used to calculate the roughness. Using this information, a kernel size of 4.3 mm was selected and the average surface roughness on the fractures generated was 0.16 (SD = 0.07) mm.

## 4 Discussion and conclusion

Previous studies have demonstrated that the healing process of fractured bone is strongly dependent on the mechanical environment at the fracture site and the forces applied to the limb by the physical activity of the patient (Kleinnulend et al., 1995). However, there has been little work in investigating the effect of the surface properties of the fracture, even though these properties will affect the relative movement of the fracture surfaces and the mechanical behaviour of the bone following fixation. As a first step, a method to repeatedly generate transverse fractures in porcine femora *in-vitro* was developed in this study along with a non-destructive approach to characterise the fracture surface geometric properties.

Several methods have been previously proposed for the generation of transverse fractures in femoral bone (Bonnarens and Einhorn, 1984; Macdonald et al., 1988; An et al., 1994; Marturano et al., 2008); however, the majority of these procedures either create fracture geometries that do not replicate those observed clinically or do not have sufficient control over the energy applied to the specimen to generate the fractures repeatedly. The drop-weight three-point bending method explained in this paper enabled repeatable fractures to be obtained in all of the specimens tested, and has the flexibility to be adapted for other bone types and impact energies. Even though the fractures generated were classified as transverse, there were a large range of geometries and topographical features of the fractured surfaces.

Interfragmentary movement (IFM) and mechanical stimuli have been found to have a strong influence in the fracture healing process and callus formation (Wu et al., 1984; Goodship and Kenwright, 1985; Claes et al., 1995; Claes et al., 1997; Claes, 2011; Meyers et al., 2017). The tissue differentiation that leads to callus formation is believed to be controlled by the distribution of local strain, which is largely dependent on mechanical factors such as fracture topography and IFM (Wang et al., 2017). In two separate a finite element studies, Mak (2013) and Leonidou et al. (2015) investigated the influence of the fracture angle on the stress generated on a locking plate used as a fracture fixation device. The results showed that the orientation of the fracture had a large effect on the fracture stabilisation, highlighting the importance of including realistic fracture geometry in computer models in order to simulate an accurate prediction of the fracture stabilisation. In finite element models, the overall fracture topography can be represented in the model geometry, and the effect of smaller scale fracture roughness by the frictional properties assigned to the fracture surface. It is therefore necessary to characterise both of these properties. Studies have measured the micro scale roughness in fractured bones using SEM by coating the surfaces with special materials (Wynnyckyj et al., 2011) with precision of the results in the region of tens of microns. However, the small size of the specimens and the need to use special coatings precludes further mechanical characterisation of these specimens.

The aim of the present study was to characterise the full fracture profile non-destructively to enable mechanical testing of the specimens in the future. The complex shape of the fractured bone was captured and characterised using a novel methodology that combined high resolution computed tomography and an in-house scripted fracture profile extraction method. It was necessary to extract these small perturbations from the overall fracture form in order to derive numerical values for the surface roughness, which was achieved using Gaussian filtering to define the form. Using artificially generated waveforms and images with different shapes and frequencies of waveform typical of those seen experimentally, the methodology employed was shown to be successful in extracting different shaped perturbations of 0.5 mm wavelength with the image resolution used in this study. The choice of kernel size used in the smoothing operation clearly affects the calculated roughness: if it is too small then the roughness is not smoothed out (Fig. 7a) and too large then the form is also smoothed (Fig. 7d). As shown in Fig. 6d, the upper bound on the kernel size above which the form itself is smoothed is affected by the form wavelength, with a kernel size greater than approximately 1/4 of the form wavelength causing the form itself to be smoothed. The form profile and number of wavelengths was quite variable between specimens in the experimental study, illustrating why the standard deviation in calculated roughness increases at larger kernel sizes (Fig. 8).

Unlike the artificially generated profiles, in reality there are a range of different wavelengths and amplitudes present in the real fracture profiles and not a simple cut-off between form and roughness. For the specimens in this study, there were not more than three large waveforms in the fracture profile, and following the conclusion from the validation study, using a kernel size of approximately 4 mm (the equivalent of nearly ¼ of the wavelength) would smooth the biggest range of roughness perturbations without adversely smoothing the form. The latter is consistent with the results presented in Fig. 8, as the selected kernel size lies in the range where the change in mean roughness is relatively low, confirming the smoothing of the roughness only. It should be noted that this roughness measurement is also linked to the resolution obtained from the CT scanner, and consideration of sampling frequency is also important when applying this type of methodology.

The methodology to generate repeatable fractures presented in this study was developed primarily to create fractures *in-vitro*, which has applications in the biomechanical evaluation of fixation devices. Further work would be needed to adapt this methodology to the generation and characterisation of fractures *in-vivo*, where there is potential to evaluate relationships between the fracture topography and bone healing. The current characterisation methodology will only quantify one circumferential line of projections, and increasing the number of concentric circular lines will give more information regarding the roughness level and any changes in the radial direction. The non-destructive nature of the approach presented in this paper, and the capacity to characterise the full bone circumference rather than a specific region, mean that there is potential to mechanically test the specimens following imaging. This opens the possibility of relating fracture surface properties to mechanical behaviour, which will be a focus of future studies.

In conclusion, in this paper a methodology for the generation of realistic fractures, characterisation and quantification of roughness of these fractures was described. The methodology presented in this manuscript can capture the realistic geometry of the fracture, which was separated into form and roughness. These parameters can be used to represent a closer fracture geometry to the ones observed clinically. This is the first step in the generation of computational models of bone that include realistic fracture geometries, and the effects of highly irregular geometries. These models will be of clinical relevance as they could be used to predict the behaviour of a variety of fracture repair devices, ultimately enabling improvements in their design for different types of fracture morphology.

## Transparency document

Transparency document Image 1
